# Why scholarly publishing might be a bubble

**DOI:** 10.3325/cmj.2017.58.1

**Published:** 2017-02

**Authors:** Hrvoje Barić, Ksenija Baždarić, Anton Glasnović, Srećko Gajović

**Affiliations:** 1 Croatian Medical Journal, University of Zagreb School of Medicine, Zagreb, Croatia, *hrvoje.baric@cmj.hr*; 2 Department of Medical Informatics, Rijeka University Faculty of Medicine, Rijeka, Croatia; 3 Croatian Institute for Brain Research, University of Zagreb School of Medicine, Zagreb, Croatia

The world's entire scientific and cultural heritage, published over centuries in books and journals, is increasingly being digitized and locked up by a handful of private corporations. -Aaron Swartz

Scholarly communication began as an interpersonal exchange of findings between fellow researchers (1). In time, the need arose for a structured, comprehensive, and pragmatic means of communicating scientific knowledge in the form of scholarly journals. Scientists did research for the sake of progress, and journals strived to catalyze the dissemination of research as widely as possible. In the age of the Internet, virtually all barriers to interpersonal communication, including scientific, have collapsed. Rather miraculously, major publishers managed to survive in the virtual environment, without suffering much harm. Not only did they survive, but they also continued to grow ever stronger, defying the new reality of a digitized, effortless scientific communication. In 1995, the Forbes magazine made a gloomy prediction - Elsevier, the largest publisher of scientific journals, would be “the Internet’s first victim“ (2). Twenty years later, in 2015, Elsevier reported an exceptionally high profit margin of 34%. Here lies the paradox: factors that justify the high costs of scholarly publishing (largely secondary to science itself) are progressively fading while, concurrently, the same new circumstances provide the necessary infrastructure for scientific communication to become cheaper, circumventing intermediaries. Still, the price of publishing services increases, and the high-quality supply is funneled to a small number of big players.

## The recipe for a bubble

Market bubbles form when a commodity gets overvalued and its price, unwarranted by intrinsic value, rises. They deflate when the self-perpetuating shared (mis)belief that a commodity will always return benefits, regardless of its cost, gets forced beyond extreme. Scientific information is considered economic commodity, and scholarly publishing behaves in entrepreneurial manner (3,4). Turning toward profit set scholarly publishing and academia on divergent independent trajectories; nevertheless, in many aspects, the two fields remain interconnected and what happens in academia is bound to reflect in scholarly publishing and vice versa. Since signs of a possible higher education bubble are looming large – the high levels of underemployment or unemployment of the highly educated might be due to overvalued academic degrees (5,6) - it would be unrealistic to rule out the possibility that scholarly publishing might follow a similar path.

## Does the publishing business follow the rules of the free market?

The workflow in scholarly publishing involves knowledge producers – scientists, consumers (universities, libraries, scientists, patients, and so on), and publishers, the latter acting as the “middle man” and providing the means of dissemination of goods (3). In comparison with other systems of exchange, the economy of publishing has many peculiarities. First, the majority of suppliers (authors-scientists) provide the product for free (likewise, the majority of peer-reviewers provide their services for free) and purchase and use are not directly connected (1). Second, both demand and supply for journals are inelastic; the rise or fall in their price is not followed by a corresponding change in demand and supply (7). Third, the commodity in question is a rare example of a public good being sold through a private market (7). Knowledge is like clean air – everyone should be allowed access to use it, and no one should reduce its availability to others. Finally, the major publishers act as an oligopoly and, occasionally, even monopoly (1). These specific circumstances enable big players to behave according to rules other than those dictated by the free market. As one science librarian put it nicely, when responding to a question why publishers charge so much for subscriptions – “because they can” (8).

## Everybody knows the emperor has no clothes, yet a few seem to care

Repeatedly, from different perspectives and in different forms, researchers and editors call attention to the following list of facts: that most research findings are false (9), only a tiny fraction of published papers in most journals meets criteria of relevance and soundness (10), physicians do not read papers (11), papers are not published to disseminate knowledge, bibliometric indicators are being misused (12), “publisher adds relatively little value to the publishing process“ (3), “the deficiencies in the editorial strategies“ are being exploited for the sake of profit (13), current practice of mass production of publications is misleading and redundant (14), it creates research waste (15), distorts science (16), and, finally, might lead to a decline of the evidence-based medicine paradigm (17). In spite of these and many other warnings, the number of publications, journals, and publishers is constantly on the rise. To illustrate, the number of articles indexed by Medline in 1975 was close to a quarter million and it doubled to half a million by 2000. It took only ten years for this figure to double again and, in 2010, there were roughly a million newly indexed articles. If this trend continues, we will probably reach two million by 2030, which means that in 15 years’ time, only in Medline, there will be over five thousand new articles daily. For most of these articles, their contribution will be to add to the cacophony and noise, not to science. Along with this growth in number of publications, and ever-louder warnings of its perils, the publishing business seems to be overcoming one obstacle after another. Subscription prices of scholarly journals have been growing faster than the consumer price index and the inflation rate (18). Between 1986 and 2001, journal prices grew more than three times as fast as the consumer price index, and two times as the health care prices (7). Having this in mind, it comes as no surprise that the major publishers have extraordinarily high profit margins.

## The inflationary nature of the publishing cosmos

The above listed arguments support the notion that scholarly publishing is expanding in all directions, like a bubble. The number of publications and rising prices are the main but not the only problem. There is also high number of publishers, journals, journals per publisher, predatory journals, authorships per article and per unique author, number of references per paper, self-citated and self-citing rates, and so on – they all relentlessly increase (19-22). Thus, the inflation might be described by a fraction with the exponential growth in the nominator and eroding value in the denominator. A plausible hypothesis is that the expansion is driven by a market bubble – the dark energy of the publishing cosmos. Still another inflationary force comes from within the academia, where bibliometric indicators govern the academic value system, which then reflects on the academic hierarchy and funding.

## Catalyzing dissemination of good science is all that matters

One of the scientific breakthroughs in 2015 was the Bell´s theorem. It is a theorem in quantum mechanics named after the physicist John Stewart Bell and it has been described as “the most profound discovery in science” (23). Apart from its scientific relevance, Bell´s theorem is an interesting case of how bad publishing practices can get into way of good science. Namely, much of his early work Bell published in *Epistemological Letters* – a “subversive” physics newsletter created because Bell and his colleagues could not publish their work in major scholarly journals (24). History is rich with cases similar to Bell`s, and they serve to remind us that good science always finds its way: sometimes due to publishing practices and sometimes in spite of them. Not all publishing is bad, far from it, but there is too much evidence to ignore that the current model might be harming science.

## The deflation

Bubbles deflate because of paradigm shifts – a radical change in the production process of goods (25). In case of scholarly publishing, a paradigm shift – the digital one – happened decades ago and yet, the bubble continued to grow. The other cause of deflation could be a change in consumer behavior. The open access (OA) movement was precisely the type of incentive expected to bring about that change. However, the publishing business continued to flourish under the gold OA, where authors were “offered” to cover the costs of publishing. It seems as the only thing that has changed is the path of the money – the cost of publishing has remained as high as before or become even higher (26). The diamond (platinum) OA, under which there are no publishing fees whatsoever, articulates the idea that the change in our behavior toward publishing is paramount if we are to preserve a relevant, sound, and unbiased science. Eventually, the publishing bubble will deflate, and the diamond OA is its promising candidate successor. The *Croatian Medical Journal*, a taxpayer-funded no-profit journal, is proud to be part of the diamond OA and it continues to provide a platform for archiving and free exchange of science. Our model serves as an example that small journals can survive in the modern digital era, without losing sovereignty or independence, and keeping a strong emphasis on research integrity. We are not saying that publishers should not get paid, but the price of profit in publishing is too high and if the current publishing practice continues, we are at risk of compromising the one thing publishing was meant to help and serve – science.
